# The methyltransferase Rrp8 is a positive regulator of autophagy flux in *Saccharomyces cerevisiae*

**DOI:** 10.17912/micropub.biology.002252

**Published:** 2026-08-03

**Authors:** Swaroopa Badenahalli Narasimhaiah, Elizabeth Delorme-Axford

**Affiliations:** 1 Biological Sciences, Oakland University, Rochester, MI, United States

## Abstract

Macroautophagy (hereafter referred to as autophagy) is a dynamic pathway of cellular degradation and recycling that is conserved from yeast to humans. The products of autophagic degradation may be used for anabolic reactions during nutrient-limited conditions. Thus, autophagy serves both metabolic and quality control functions. However, the role of nucleolar proteins in autophagy remains largely unexplored. Here we identify Ribosomal RNA processing 8 (Rrp8) as a positive regulator of autophagy flux in the yeast *Saccharomyces cerevisiae.* Our work provides insight into the role of the conserved nucleolar protein Rrp8 in regulating cellular responses to starvation.

**Figure 1. Rrp8 positively regulates autophagy flux:** ( **A** ) WT (JMY347), *atg13* Δ (EDA328), and *rrp8* Δ (SBN08) strains were grown to mid-log phase in YPD (+N), then starved for nitrogen (-N) in SD-N medium for 4 h. Pho8Δ60 activity was measured and normalized to the activity of starved WT cells, which was set at 100%. ( **B** ) WT (EDA291) and *rrp8* Δ (SBN03) strains expressing GFP-Atg8 were grown in nutrient-rich media, and then starved for nitrogen as indicated *.* Protein extracts were analyzed by SDS-PAGE and blotted using an antibody that recognizes GFP. GAPDH is the loading control. A representative blot is shown (n=3). ( **C** ) Densitometry of blots represented in ( **B** ). Processed GFP-Atg8 was calculated by determining the ratio of free GFP:total GFP (sum of free GFP and full-length GFP-Atg8). Results shown are relative to the WT strain during starvation (2 h SD-N), which was set to 1 (n=3). ( **D** ) Loss of *RRP8* decreases cell survival after prolonged nitrogen starvation. WT (BY4742) and *rrp8* ∆ cells were grown in nutrient-rich YPD medium (+N) until mid-log phase and then starved for nitrogen for 7 days (–N). Cells were serially diluted and spotted onto YPD plates, incubated for 2 days, and imaged. The image shown is representative of n=4 independent experiments. ( **E** ) *RRP8* expression decreases during nitrogen starvation. WT (BY4741) cells were grown to mid-log phase in YPD (+N) and then shifted to nitrogen starved (-N) media for 1 hour. Total RNA was extracted, and RT-qPCR was performed. Results shown are relative to the level of expression in WT cells under rich conditions, which was set to 1 (n=3). ( **F** ) Rrp8-PA fusion protein levels decrease with nitrogen starvation. WT (SBN15) cells endogenously expressing Rrp8-PA fusion protein were grown to mid-log phase in YPD then starved for nitrogen for the time points indicated. Protein extracts were resolved by SDS-PAGE and blotted with anti-PA or anti-GAPDH (loading control) antibodies. A representative blot is shown (n=4). ( **G** ) Densitometry of blots represented in ( **F** ). The percentage of Rrp8-PA:GAPDH was quantified. ( **H** ) Loss of *SFP1* decreases Rrp8-PA fusion protein levels. WT (SBN15) and *sfp1* Δ (SBN21) endogenously expressing Rrp8-PA were grown to mid-log phase in YPD (+N) starved for nitrogen for 2 h (-N). Protein extracts were analyzed as in ( **F** ). A representative blot is shown (n=3). ( **I** ) Densitometry of blots represented in ( **H** ). The percentage of Rrp8-PA:GAPDH was quantified. For ( **A** ), ( **C** ), ( **E** ), ( **G** ), and ( **I** ), results shown are the mean. Error bars indicate standard deviation.

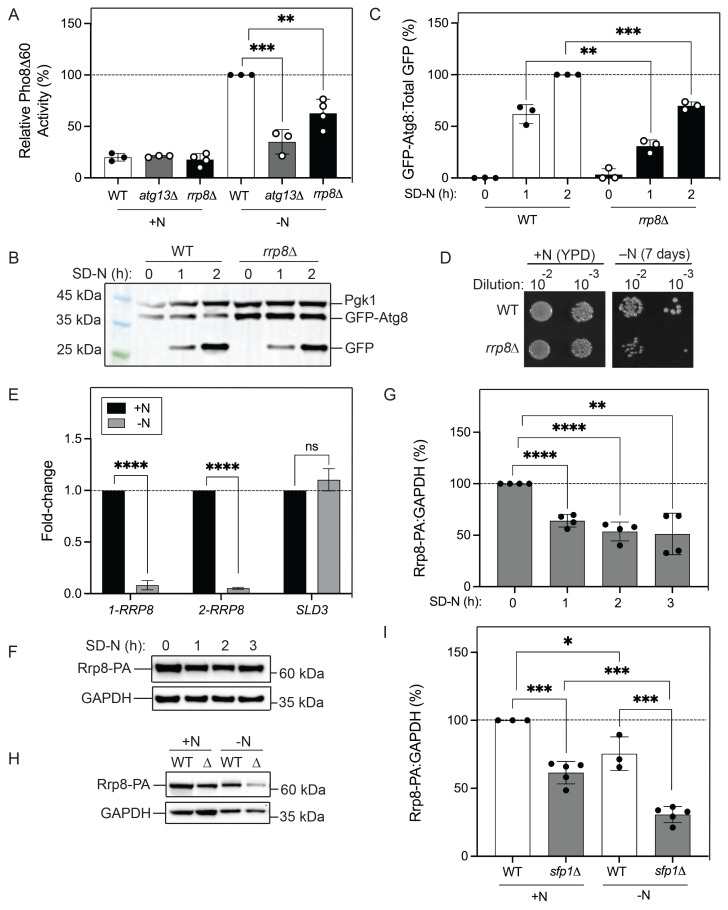



## Description

Macroautophagy (hereafter referred to as autophagy) is a dynamic pathway of cellular degradation and recycling that is conserved from yeast to humans. Nonselective autophagy targets bulk cytoplasm; whereas selective forms of autophagy target specific intracellular cargo. Basal autophagy is low, but is markedly upregulated during stressfulconditions such as nutrient deprivation. Canonically, autophagy is a catabolic process, breaking down cytoplasmic cargo within the vacuole (in yeast) to maintain cell survival. The autophagic cargo are degraded, and the resulting biomolecules are transported to the cytosol for reuse. The effluxed bioproducts of autophagic degradation can be used for anabolic reactions during nutrient-limited conditions. Thus, autophagy serves both metabolic and quality control functions.

The goal of this study was to investigate the role of the budding yeast *Saccharomyces cerevisiae* ribosomal RNA processing 8 (Rrp8) protein in autophagy. Rrp8 is a S-adenosylmethionine-dependent methyltransferase (Bousquet-Antonelli et al., 2000). Rrp8 methylates m ^1^ A645 of 25S rRNA (Peifer et al., 2013). The N ^1^ -methyladenosine (m ^1^ A) modification is important for regulating gene expression (Li et al., 2022). m ^1^ A is a reversible modification in tRNA, mRNA, rRNA and long non-coding RNA (lncRNA) and has impacts on RNA processing, structure, and functions of targets (Li et al., 2022). Nucleomethylin (the mammalian homolog of yeast Rrp8) is associated with metabolic disease, obesity, and nutrient availability signaling (Oie et al., 2014; Sharma et al., 2018). Additionally, there is a gap in our understanding of how autophagy contributes to cellular metabolism, and in return, how metabolism impacts autophagy activity.

Given the relationship between nucleomethylin and nutrient availability signaling, we examined whether Rrp8 (the yeast homolog of nucleomethylin) plays a role in autophagy in *Saccharomyces cerevisiae.* To the best of our knowledge, whether Rrp8 has any impact on autophagy– in any model system– has never been explored. We first investigated whether autophagy was affected in cells lacking *RRP8* by performing the alkaline phosphatase (ALP) or Pho8Δ60 assay ( Figure 1A ). The ALP assay is a quantitative enzymatic measurement of autophagy (Noda & Klionsky, 2008), although some minimal degree of basal phosphatase activity is observed in cells under nutrient-rich conditions (Delorme-Axford et al., 2018). As expected, when cells were starved for nitrogen (4 h), robust autophagy activity is observed in the wild-type (WT) strain and little activity in the negative control *atg13* ∆ strain (~35%; Figure 1A ). Atg13 is essential for autophagy in yeast (Tsukada & Ohsumi, 1993). Pho8Δ60 activity is reduced in the *rrp8* ∆ strain compared to WT (~63%; Figure 1A ), supporting that autophagy flux decreases in cells lacking *RRP8* .

Based on these results, we next examined whether autophagy was impaired in *rrp8* Δ cells by performing the GFP-Atg8 processing assay ( Figure 1B, C). The GFP-Atg8 assay is a method to monitor bulk autophagy progression based on the release of free GFP. Following autophagosome-vacuole fusion, Atg8 is rapidly hydrolyzed; the GFP moiety remains relatively stable within the vacuole (Cheong & Klionsky, 2008). The release of free GFP from the GFP-Atg8 fusion protein indicates autophagy flux following autophagosome-vacuole fusion (Delorme-Axford et al., 2015). Consistent with our Pho8Δ60 assay results ( Figure 1A ), we observed a significant decrease of free GFP release in the *rrp8* Δ cells compared to the WT at 1 and 2 h of nitrogen starvation ( Figure 1B, C). To determine whether loss of *RRP8* impacts cell survival, we examined WT and *rrp8* ∆ cells under nutrient-rich and prolonged nitrogen starvation conditions ( Figure 1D ). Following 7 days of nitrogen starvation, *rrp8* Δ cells showed reduced survival compared to WT, suggesting that Rrp8 may play an important role in mediating cell survival under nutrient-stress conditions ( Figure 1D ). This is consistent with previous observations that cells deficient in autophagy display reduced viability under prolonged starvation (Bernard, Jin, González-Rodríguez, et al., 2015; Tsukada & Ohsumi, 1993). Taken together, these data support the idea that Rrp8 is a positive regulator of autophagy flux when cells are starved for nitrogen ( Figure 1A –D).

To determine whether *RRP8* expression changes during autophagy-inducing conditions, we assessed *RRP8* mRNA levels using RT-qPCR ( Figure 1E ). Two pairs of independent primers ( *1-RRP8* and *2-RRP8* ) were used to ensure reliability ( Figure 1E ). Following nitrogen starvation, *RRP8* expression markedly declined by 1 hour of starvation (>90%; Figure 1E ). As a control, *SLD3* (a gene with no known connection to autophagy) was also analyzed ( Figure 1E ). As expected, *SLD3* expression did not significantly change between nutrient-rich and starvation conditions ( Figure 1E ). To monitor Rrp8 protein levels, *RRP8* was chromosomally tagged at the C-terminus with Protein A (PA), allowing detection of endogenous Rrp8-PA fusion protein during a time course of nitrogen starvation (0, 1, 2, and 3 h; Figure 1F, 1G). Western blot analysis revealed a noticeable decrease in Rrp8-PA protein levels during nitrogen starvation compared to nutrient-rich conditions, suggesting that Rrp8 is downregulated during nutrient stress (~50% by 3 h; Figure 1F, 1G).

The split-finger protein 1 (Sfp1) is a nutrient and stress sensitive transcription factor (Albert et al., 2019). Sfp1 is an activator of ribosomal protein and ribosome biogenesis gene transcription in yeast (Marion et al., 2004). Sfp1 is phosphorylated by the Target of Rapamycin Complex 1 (TORC1) kinase at multiple sites (Lempiäinen et al., 2009). Tor kinase is a negative regulator of autophagy (Noda & Ohsumi, 1998). Rapamycin treatment strongly inhibits the TORC1-Sfp1 interaction, leading to Sfp1 dephosphorylation (Lempiäinen et al., 2009). Rapamycin inhibits Tor, thereby activating autophagy (reviewed in (Delorme-Axford et al., 2015)). In addition, prior work by others suggests that Sfp1 may be a potential transcriptional activator of *RRP8* (Cipollina et al., 2008). To investigate whether Sfp1 regulates *RRP8* , we compared Rrp8-PA fusion protein levels in WT and *sfp1* Δ cells by western blot analysis ( Figure 1H, 1I). Under nutrient-rich conditions (+N), Rrp8-PA is well expressed in WT cells and decreases when cells are starved for nitrogen (-N; Figure 1H, 1I). In contrast, Rrp8-PA levels were reduced in *sfp1* Δ cells relative to WT under both nutrient-rich and starved conditions ( Figure 1H, 1I). Taken together, these data suggest that Sfp1 may function as a positive regulator of Rrp8.

Here we characterize the yeast methyltransferase Rrp8 as a positive regulator of autophagy flux. Although we observed that *RRP8* /Rrp8 levels decline during nitrogen starvation, we do not currently know the mechanism(s) underlying our observations and/or what role the enzymatic activity of Rrp8 may have in this process. It is interesting to speculate that the observed decrease in *RRP8* /Rrp8 levels could serve as a mechanism to limit enzyme activity. We also found that loss of TORC1-regulated transcription factor *SFP1* decreases Rrp8 levels under nutrient-rich and starved conditions, suggesting that Sfp1 may regulate Rrp8 expression. This work provides insight into the role of the conserved nucleolar protein Rrp8 in regulating cellular responses to starvation. Further elucidation of these mechanisms will advance our understanding of the molecular crosstalk between ribosome biogenesis, metabolism, and autophagy.

## Methods

***Yeast Strains, Media, and Cell Culture:*** *Saccharomyces cerevisiae***** yeast strains used in this study are listed in the accompanying table. Yeast cells were grown in YPD (1% yeast extract, 2% peptone, and 2% glucose) medium (Gibco, A1374501). To induce autophagy, cells were grown to mid-log phase in YPD, then shifted to**** nitrogen starvation medium (SD-N; 0.17% yeast nitrogen base without ammonium sulfate or amino acids and 2% glucose) for the indicated time points. Chromosome tagging and gene deletions were performed using established methods (Gueldener et al., 2002; Longtine et al., 1998).

***Pho8Δ60 Assay:*** The Pho8Δ60 assay was performed using the SmartReader 96 microplate absorbance reader (Accuris, MR9600) as previously described (Tasmi et al., 2026). Pho8Δ60 values were normalized to the protein content of each sample determined by the Pierce BCA Protein Assay kit (Thermo Scientific, 23227) as described (Delorme-Axford et al., 2023).

***SDS-PAGE and Western Blots:*** SDS-PAGE and western blots were performed as previously described (Tasmi et al., 2026). Western blots were visualized using the Azure 600 (Azure Biosystems) or iBright CL1500 (Invitrogen) imaging systems. Densitometry for western blots was performed using ImageJ ( https://imagej.nih.gov/ij/ ). Antibodies used in this study are included in the accompanying table.

***Yeast Viability Assay:*** Yeast growth assays were performed as previously described (Avogo et al., 2025).

***RNA and Real-Time Quantitative PCR (RT-qPCR):*** RNA extraction and RT-qPCR was performed as previously described (Avogo et al., 2025). Relative gene expression was calculated using the 2 ^−ΔΔCT^ method (Livak and Schmittgen 2001), normalized to *UBC6* levels. RT-qPCR primers used in this study are listed in the accompanying table.

***Statistical analysis:*** The two-tailed unpaired *t* test was used to determine statistical significance with GraphPad Prism (GraphPad Software, USA). For Figure 1, *p* values are as follows: * *p* <0.05; ** *p* <0.01; *** *p* <0.001; **** *p* <0.0001; ns indicates not significant. A *p* value < 0.05 was considered significant.

## Reagents

**Table:** 

***Saccharomyces cerevisiae* strains used in this study are as follows: **		
**Strains**	**Genotype**	**Reference**
BY4741	*MATα* *his3* Δ *1* *leu2* Δ *0* *met15* Δ *0* *ura3* Δ *0*	Horizon Discovery
BY4742	*MATα* *his3* Δ1 *leu2* Δ0 *lys2* Δ0 *ura3* Δ0	Horizon Discovery
BY4742, *rrp8* Δ *::KANMX*	BY4742, *rrp8* Δ *::KANMX*	Horizon Discovery
EDA291	WLY176, *CUP1p-GFP-ATG8(405)::LEU2*	This study
EDA328	JMY347, *atg13* Δ:: *HIS5*	(Tasmi et al., 2026)
JMY347	SEY6210, *pho13* Δ *ZEO1p-pho8* Δ *60, CUP1p-GFP-ATG8(405)::LEU2*	(Wen et al., 2020)
SBN03	EDA291, *rrp8* Δ *::URA3*	This study
SBN08	JMY347, *rrp8* Δ:: *HIS5*	This study
SBN15	BY4742, *RRP8-PA::HIS3*	This study
SBN21	SBN15, *sfp1* Δ:: *URA3*	This study
SEY6210	MATα *leu2-3,112 ura3-52 his3-* Δ *200 trp1-* Δ *901 suc2-* Δ *9 lys2-801; GAL*	(Robinson et al., 1988)
WLY176	SEY6210, *pho13* ∆ *pho8::pho8* ∆ *60*	(Kanki et al., 2009)
**RT-qPCR primer sequences used in this study are as follows:**		
			**Primer**	**Sequence (5' to 3')**	**Reference**
*1-RRP8-qPCR-F*	ACGCTTTGAAGCTGATGGGA	This study
*1-RRP8-qPCR-R*	TAATCTCCGCAGGTGGCTTG	This study
*2-RRP8-qPCR-F*	TTTAGCGCCAAGGGGTGAAT	This study
*2-RRP8-qPCR-R*	CCCATCAGCTTCAAAGCGTC	This study
*SLD3-qPCR-F*	CGCAACTTCAAAGCATCATTGAATCGC	(Bernard, Jin, Xu, et al., 2015)
*SLD3-qPCR-R*	GGGGCTTATTAGTGGGAGTAGAGG	(Bernard, Jin, Xu, et al., 2015)
*UBC6-qPCR-F*	GATACTTGGAATCCTGGCTGGTCTGTCTC	(Teste et al., 2009)
*UBC6-qPCR-R*	AAAGGGTCTTCTGTTTCATCACCTGTATTTGC	(Teste et al., 2009)
**Antibodies used in this study are as follows:**		
**Name **	**Identifier (Source)**	**Concentration**
mouse monoclonal anti-GAPDH (1E6D9)	Cat# 60004-1-Ig (Proteintech)	1:20,000
mouse monoclonal anti-GFP (JL-8)	Cat# 632381 (Clontech)	1:3,000
rabbit polyclonal anti-peroxidase (anti-PA) antibody	Cat# 323-005-024 (Jackson Immunoresearch)	1:30,000
mouse monoclonal anti-Pgk1 (22C5D8)	Cat# 459250 (Invitrogen)	1:5,000
